# Effectiveness of an intervention for improving drug prescription in primary care patients with multimorbidity and polypharmacy: study protocol of a cluster randomized clinical trial (Multi-PAP project)

**DOI:** 10.1186/s13012-017-0584-x

**Published:** 2017-04-27

**Authors:** Alexandra Prados-Torres, Isabel del Cura-González, Daniel Prados-Torres, Juan A. López-Rodríguez, Francisca Leiva-Fernández, Amaia Calderón-Larrañaga, Fernando López-Verde, Luis A. Gimeno-Feliu, Esperanza Escortell-Mayor, Victoria Pico-Soler, Teresa Sanz-Cuesta, Mª Josefa Bujalance-Zafra, Mariel Morey-Montalvo, José Ramón Boxó-Cifuentes, Beatriz Poblador-Plou, José Manuel Fernández-Arquero, Francisca González-Rubio, María D. Ramiro-González, Carlos Coscollar-Santaliestra, Jesús Martín-Fernández, Mª Pilar Barnestein-Fonseca, José María Valderas-Martínez, Alessandra Marengoni, Christiane Muth, Multi-PAP Group, Mercedes Aza-Pascual-Salcedo, Marta Alcaraz-Borrajo, José Mª Ruiz-San-Basilio, Ángel Mataix-San-Juan, Ana Mª López-León, Antonio Poncel-Falcó, Carmina Mateos-Sancho, Antonio Gimeno-Miguel, Virginia Hernández-Santiago, Ana Cristina Bandrés-Liso, Francisca García de- Blas, Nuria García-Agua, Ricardo Rodríguez-Barrientos, Rubén Vázquez-Alarcón, Clara Laguna-Berna, Maria Isabel Márquez-Chamizo, Javier Marta-Moreno, Amaya Azcoaga-Lorenzo, José María Abad-Díez, Luis Sánchez-Perruca, Elena Polentinos-Castro, Mercedes Clerencia-Sierra, Gloria Ariza-Cardiel, Ana Isabel González-González, Milagros Rico-Blázquez, Marisa Rogero-Blanco, Mª Eugenia Tello-Bernabé, Mar Álvarez-Villalba, Mercedes Rumayor-Zarzuelo

**Affiliations:** 1 Instituto Aragonés de Ciencias de la Salud (IACS), IIS Aragón, Hospital Universitario Miguel Servet, Paseo Isabel La Católica 1-3, 50009 Zaragoza, Spain; 2 Departamento de Microbiología, Medicina Preventiva y Salud Pública, Universidad de Zaragoza, Zaragoza, Spain; 3 Red de Investigación en Servicios de Salud en Enfermedades Crónicas (REDISSEC) ISCIII, Madrid, Spain; 4 Joint Action on Chronic Diseases (JA-CHRODIS) European Comission, Brussels, Belgium; 5 Unidad de Apoyo a la Investigación, Gerencia Asistencial de Atención Primaria, Servicio Madrileño de Salud, Madrid, Spain; 6 Área Medicina Preventiva y Salud Pública. Facultad de Ciencias de la Salud, Universidad Rey Juan Carlos, Madrid, Spain; 7 Unidad Docente Multiprofesional de Atención Familiar y Comunitaria de Distrito Málaga/Guadalhorce, Málaga, Spain; 8 Instituto de Investigación Biomédica de Málaga (IBIMA), Distrito Sanitario Málaga/Guadalhorce, Málaga, Spain; 9 Universidad de Málaga, Málaga, Spain; 10 Centro de Salud General Ricardos, Madrid, Spain; 11 Karolinska Institutet, Aging Research Center, Stockholm, Sweden; 12 Centro de Salud las Delicias, Distrito Sanitario Málaga/Guadalhorce, Málaga, Spain; 13 Centro de Salud San Pablo, Zaragoza, Spain; 14 Centro de Salud Torrero-La Paz, Zaragoza, Spain; 15 Centro de Salud la Victoria, Distrito Sanitario Málaga/Guadalhorce, Málaga, Spain; 16 Centro de Salud Puerta Blanca, Distrito Sanitario Málaga/Guadalhorce, Málaga, Spain; 17 Servicio de Farmacia de Atención Primaria, Distrito Sanitario Málaga/Guadalhorce, Málaga, Spain; 18 Centro de Salud Delicias Sur, Zaragoza, Spain; 19 Servicio de Medicina Preventiva, Hospital Universitario Gregorio Marañón, Madrid, Spain; 20 Centro de Salud de Villamanta, Madrid, Spain; 21 University of Exeter Medical School, Exeter, UK; 22 Department of Clinical and Experimental Sciences, University of Brescia, Brescia, Italy; 23 Institute of General Practice, Johann Wolfgang Goethe University, Frankfurt, Germany

## Abstract

**Background:**

Multimorbidity is associated with negative effects both on people’s health and on healthcare systems. A key problem linked to multimorbidity is polypharmacy, which in turn is associated with increased risk of partly preventable adverse effects, including mortality. The Ariadne principles describe a model of care based on a thorough assessment of diseases, treatments (and potential interactions), clinical status, context and preferences of patients with multimorbidity, with the aim of prioritizing and sharing realistic treatment goals that guide an individualized management. The aim of this study is to evaluate the effectiveness of a complex intervention that implements the Ariadne principles in a population of young-old patients with multimorbidity and polypharmacy. The intervention seeks to improve the appropriateness of prescribing in primary care (PC), as measured by the medication appropriateness index (MAI) score at 6 and 12 months, as compared with usual care.

**Methods/Design:**

Design: pragmatic cluster randomized clinical trial. Unit of randomization: family physician (FP). Unit of analysis: patient. Scope: PC health centres in three autonomous communities: Aragon, Madrid, and Andalusia (Spain). Population: patients aged 65–74 years with multimorbidity (≥3 chronic diseases) and polypharmacy (≥5 drugs prescribed in ≥3 months). Sample size: *n* = 400 (200 per study arm). Intervention: complex intervention based on the implementation of the Ariadne principles with two components: (1) FP training and (2) FP-patient interview. Outcomes: MAI score, health services use, quality of life (Euroqol 5D-5L), pharmacotherapy and adherence to treatment (Morisky-Green, Haynes-Sackett), and clinical and socio-demographic variables. Statistical analysis: primary outcome is the difference in MAI score between T0 and T1 and corresponding 95% confidence interval. Adjustment for confounding factors will be performed by multilevel analysis. All analyses will be carried out in accordance with the intention-to-treat principle.

**Discussion:**

It is essential to provide evidence concerning interventions on PC patients with polypharmacy and multimorbidity, conducted in the context of routine clinical practice, and involving young-old patients with significant potential for preventing negative health outcomes.

**Trial registration:**

Clinicaltrials.gov, NCT02866799

**Electronic supplementary material:**

The online version of this article (doi:10.1186/s13012-017-0584-x) contains supplementary material, which is available to authorized users.

## Background

Multimorbidity, the presence of various chronic diseases in the same individual, is the norm among the elderly population and very prevalent in the adult population in most Western European countries [1–3]. In Spain, the latest National Health Survey reports that individuals of over 75 years of age have an average of 3.2 chronic health problems, while the so-called young-old population (65–74 years) has an average of 2.8. Most studies define multimorbidity as the concurrent presence of two or more or three or more chronic diseases; the latter definition is more suitable for the identification of patients with complex health needs [4].

The potential negative health impacts of multimorbidity include reduced quality of life and functional capacity, inadequate use of health services, and increased complications and healthcare costs [5–7]. These effects are partly attributable to the current model of healthcare, which is essentially organized and designed to address diseases individually [8–10].

Although there is emerging evidence to support policy for the management of people with multimorbidity, the effectiveness of interventions is still uncertain [11], as is the case for clinical practice guidelines (CPGs) for patients with comorbidity [12]. The reality is that uncritical application of the recommendations of multiple CPGs for concurrent diseases in the same patient increases the likelihood of polypharmacy, defined by consensus as the simultaneous consumption of five or more drugs [13]. Family physicians (FP) have reported that this is a daily reality in primary care (PC) [14].

Polypharmacy implies an increased risk of medication-related problems such as interactions and adverse drug reactions, underuse of necessary treatments, low adherence, and partly preventable mortality, in particular in older patients [15]. Inappropriate choice of drugs with regard to age is another major problem, for which alternative (safer) approaches have been proposed that are equally or more effective [16].

Multiple approaches have been designed to measure and reduce inappropriate prescribing [17]. Explicit measures assess prescriptions according to predefined criteria related to the properties of the drugs concerned (e.g. Beers [18] and STOPP/START criteria [19]). However, these criteria may fall short in patients with multiple diseases and (interacting) treatments [20]. Therefore, implicit measures are applied to determine the level of appropriateness of prescribing. Based on the clinical judgment of the rater, implicit measures take into account the health status of the individual patient. The implicit method that is most accepted and validated, both internationally and in Spain, is the medication appropriateness index (MAI) [21, 22].

Evidence supporting the effectiveness of interventions to improve outcomes in patients with multimorbidity remains scarce [8, 23]. The Cochrane systematic review by Smith et al. [24] concluded that drug prescribing and adherence tend to improve when interventions target risk factors of multimorbidity and when they focus on key issues or specific functional difficulties affecting patients. Another Cochrane systematic review [25] evaluating the effectiveness of interventions aimed at minimizing the negative effects of polypharmacy concluded that, despite an overall improvement in prescribing by physicians, the effect on other clinical variables such as hospital admissions and quality of life is unclear. For this reason, the authors emphasized the need to incorporate into clinical trial outcome variables of relevance both for clinicians and patients and to evaluate intervention costs.

Various care programmes have been implemented in Spain to address polypharmacy in elderly patients (>75 years) by means of a systematic medication review in PC, and this strategy is supported by some evidence [26]. However, patients over 75 years of age constitute only part of the population with multimorbidity. Furthermore, these strategies do not meet the requirements of patient-centred care where patient’s preferences are taken into consideration. Shared decision making between health professionals and patients is also thought to improve patient’s adherence [27].

The sharing of common and realistic treatment goals between physician and patient is essential to tackle multimorbidity in the PC context and is the cornerstone of the Ariadne principles [28]. The implementation of these principles is based on a thorough assessment of the diseases, treatments and potential treatment interactions, global clinical status, and context of the patient by the physician. This allows prioritization of patients’ health problems, taking into account their preferences and wishes and ensuring their individualized management and monitoring. Despite numerous studies demonstrating the effectiveness of shared decision making on health outcomes [29–31], the feasibility of implementation and the impact of the Ariadne principles in PC have not been assessed to date, although the potential benefit of implementing such a strategy in routine clinical practice has been recognized [32, 33].

### Objectives

#### Primary

The main objective is to evaluate the effectiveness of a complex PC intervention implementing the Ariadne principles on the improvement of medication appropriateness in the young-old population with polypharmacy and multimorbidity, as measured by the differences of the MAI score at 6 months (T1) to baseline, compared with usual care.

#### Secondary

- To evaluate the effects of the complex intervention on medication appropriateness after 12 months (T2), as well as on the use of health services, patient quality of life, treatment adherence, and medication safety, as compared with usual care.
- To study the cost-utility ratio of the intervention as compared with that of usual care.

## Method/design

### Design

Pragmatic cluster randomized controlled clinical trial with 12 months of follow-up. The unit of randomization is the FP and the unit of analysis is the patient. A cost-utility study will be performed from the perspective of the funder with a time horizon of 1 year.

### Scope of study

The scope of the study is the primary care setting of the Spanish national health system.

### Study population

The study population includes patients aged 65 to 74 years with multimorbidity and polypharmacy, attending PC health centres in three autonomous communities (ACs) in Spain: Aragon, Madrid, and Andalusia.

#### FP selection criteria

- Employed in current position for at least 1 year.
- Stable employment situation, with no intention of leaving their position during the course of the study.
- Agree to participate and provide written informed consent.

#### Patient selection criteria

1. Inclusion criteria:
  Age 65 to 74 years.
  Multimorbidity, defined as ≥3 chronic diseases as per O’Halloran [34].
  Polypharmacy, defined as ≥5 drugs prescribed over at least the 3 months prior to inclusion in the study.
  At least one visit to the FP in the past year.
  Agree to participate and provide written informed consent.
2. Exclusion criteria:
  Institutionalized patients.
  Life expectancy of less than 12 months, as determined by the FP.
  Mental and/or physical conditions considered by the FP to prevent fulfilment of study requirements.

### Sample size

The sample size was calculated under the hypothesis that the intervention would lead to a difference of at least 2 units in the change in MAI score at 6 months (T1 vs T0) (i.e. a clinically relevant difference) between study groups. Differences in MAI are assumed to be normally distributed in each intervention arm and the variances are assumed to be equal. According to previous studies, the standard deviation of the difference in MAI is 6 units [32–34]. Therefore, the study should be capable of detecting an effect size of 0.3 (2/6). Considering a power of 80% and assuming simple random sampling, the required sample size would be 286 patients (143 patients in the intervention group and 143 in the control group).

The effective sample size in this type of study design depends on the average size of the cluster and the degree of correlation between individuals in the cluster. Accordingly, it is necessary to adjust the calculated sample size in accordance with the design effect (DE). An average cluster size of 5 patients per FP and an intraclass correlation coefficient of 0.03 [35] produces the following (DE = 1 + (5 − 1) × 0.03 = 1.12) which gives a sample size, corrected for the DE, of 320 patients. Assuming a percentage of losses of 20%, the final sample size required is 400 patients (200 per group). Assuming that each FP will recruit 5 patients, 80 FPs (40 per group) will be required. In each AC, 133 patients will be recruited.

### Recruitment

Voluntary participation will be proposed to FPs working in PC health centres in each of the three ACs. Patients will be added to a randomly ordered list of potential participants provided that they fulfil the inclusion criteria. Strategies to improve protocol adherence of FP will be considered (e.g. individual follow-up of protocol’s achievements and recognition via e-mail, offer to participate as co-authors in scientific papers, certified training sessions).

**Fig. 1:**
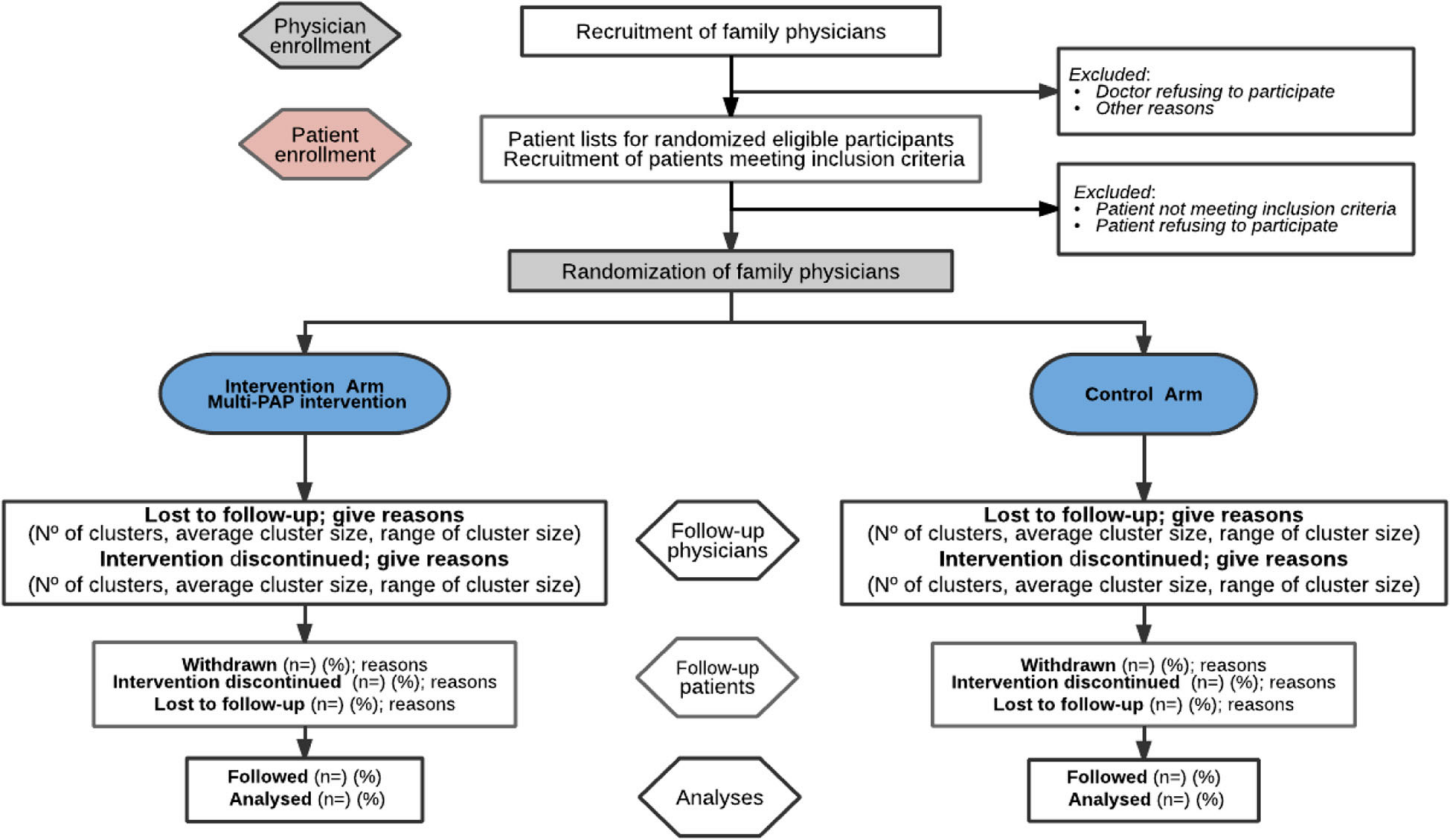
Flow diagram

Each FP will consecutively select 5 patients from this list. When a patient agrees to participate, the FP will provide them with detailed information about the study, confirm the inclusion/exclusion criteria, and obtain the patient’s written informed consent. If they do not agree to participate, data on the patient’s age, sex, and reason for nonparticipation will be collected (see Fig. 1).

### Randomization

The unit of randomization is the FP and the unit of analysis the patient. Randomization of the FP will be achieved using the treatment assignment module of the Epidat 4.1 program; the proposed intervention will be considered the treatment and usual care considered the control. To ensure an equal number of FPs in each group (intervention and control), the ‘balanced groups’ option will be selected.

Once all participating FPs have selected their patients and collected the corresponding baseline data, FP randomization will be performed centrally by the Unidad de Apoyo a la Investigación, Gerencia Asistencial de Atención Primaria in Madrid. Subsequently, each FP will receive the information on the study group to which they have been assigned, at which point all patients recruited by him or her will be included in that group.

### Intervention

#### Intervention group

A complex intervention with two phases is conducted:

- First phase: FP training. This will consist of a previously designed training activity, delivered using the massive online open courses (MOOC) format, including basic concepts relating to multimorbidity, appropriateness of prescribing, treatment adherence, the Ariadne principles, and physician-patient shared decision making.
- Second phase: Physician-patient interview based on the Ariadne principles.

**Fig. 2:**
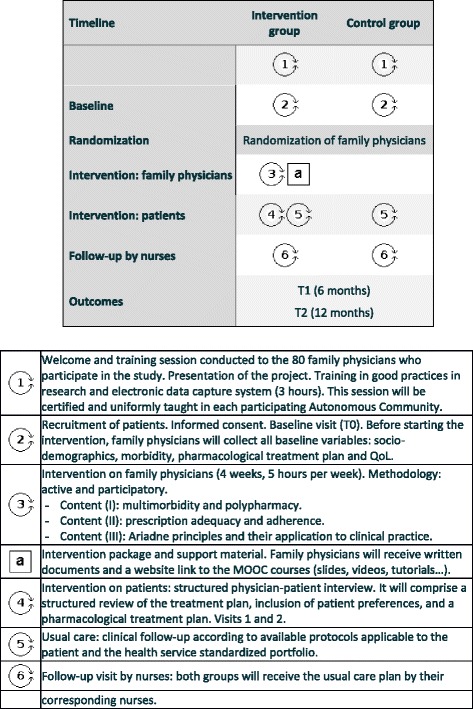
Complex intervention pat plot. *QoL* quality of life, *MOOC* Massive Online Open Courses

This intervention has been developed in accordance with the recommendations and taxonomy proposed by the Cochrane Effective Practice and Organisation of Care Review Group (EPOC). The intervention is described in detail in Fig. 2, following the approach proposed by Perera et al. [36].

#### Control group

Patients in the control group will receive usual clinical care based on the provision of advice and information and will undergo examinations as recommended in the CPGs corresponding to each of the patient’s chronic diseases.

### Variables

FPs will provide their data before the start of the study. Patient data will be collected by the recruiting FP, who will also be responsible for patient follow-up. All information will be recorded in a case report form designed for the study. Each FP will access the form from his/her personal computer via the project website using a personal identification code. Three visits are defined for patient data collection: baseline (T0), 6 months (T1), and 12 months (T2) (see Table 1).

**Table 1:** Visit plan

	T0 (baseline)		T1 (6 months)	T2 (12 months)	Responsible entity
Confirm inclusion/exclusion criteria	X				FP
Written informed consent	X				FP
Socio-demographic variables	X				FP
Morbidity variables and drug treatment plan	X		X	X	FP
Randomization of FPs		X			RU
FP intervention (intervention group)		X			RT
Patient intervention (intervention group)		X	X		FP
Use of health services	X		X	X	RT
Medication adherence	X		X	X	FP
Medication safety			X	X	FP
Quality of life	X		X	X	FP
Costs				X	RT
MAI	X		X	X	EE EC

*FP* family physician, *RU* research unit, *RT* research team, *EE* external evaluator, *EC* evaluation committee, *MAI* medication appropriateness index

#### Primary outcome

Appropriateness of prescription will be measured by the medication appropriateness index (MAI). The main outcome variable will be evaluated by an independent FP with training in the MAI. To ensure consistent ratings, an analysis of intra-observer and inter-observer reliability of evaluators will be conducted. Additionally, a FP and a pharmacist will conduct a second appraisal of the inter-observer reliability over a randomly selected 10% of the completed questionnaires. This measure has been proposed in other studies using the MAI [37].

#### Secondary outcomes

- Use of health services: unplanned and/or avoidable hospitalizations, use of emergency services and PC (FP and nurse).
- Quality of life: measured using the EuroQol 5D-5L questionnaire [38, 39].
- Medication safety: measured as the incidence of adverse drug reactions and potentially hazardous interactions, classified using the taxonomy proposed by Otero-López [40].
- Treatment adherence: measured using the Morisky-Green test [41] and the Haynes-Sackett questionnaire [42].
- Patient perception of shared decision making: measured using a single, multiple choice question, formulated ad hoc.
- Cost-utility: time spent on training FPs, cost of teaching staff, time spent on physician-patient interviews, utilities measured using the EuroQol 5D-5L.

### Explanatory and adjustment variables

#### Patient (first level) variables

- Socio-demographics: age, sex, nationality, AC of residence, marital status, socioeconomic status (monthly salary expressed as multiples of the minimum wage), family composition (number of people living at home), housing indicators, social support (Dukes-UNC-11 questionnaire adapted to Spanish [43]), profession, and social class [44].
- Morbidity: number and description of chronic diseases based on the International Classification of Diseases in PC (ICPC).
- Pharmacotherapeutic treatment plan: number and type of drugs prescribed, active ingredient, and dose of each drug.

#### FP (second level) variables

- Socio-demographics: age, sex.
- Professional performance: years of professional experience, tutor of residents (yes/no), average workload measured as the average number of daily consultations per FP during the year previous to the start of the study.
- Prior training: in polypharmacy, multimorbidity, and/or shared decision making.

### Statistical analysis

All analyses will be carried out in accordance with the intention-to-treat principle, with significance set at *p* < 0.05.

Description of baseline characteristics (qualitative and quantitative variables) of patients and professionals for each arm of the study, with corresponding 95% confidence interval (95% CI). Description of patients that abandon the study, including patient characteristics and reasons for loss during follow-up.

Basal comparison between groups using statistical tests for independent samples (Student’s *t* test or chi-square test). Tests for related samples (ANOVA for repeated measures) will be used to analyse changes within groups and between visits.

Analysis of main effectiveness: between-group difference in T1-T0 MAI score, with corresponding 95% CI. Multilevel analysis will be used to adjust models. Difference in MAI score will be considered the dependent variable; baseline patient (first level) and FP (second level) variables and treatment arm will be considered fixed-effect independent variables; and grouping by FP will be considered a random factor. Missing data pertaining to professionals and/or patients will be addressed by replacing missing values with the most recent available or baseline data.

Analysis of secondary effectiveness (non-confirmatory): between-group difference in means or proportions of T2-T0 MAI score will be determined using the appropriate statistical tests and an explanatory model will be adjusted using the same methodology applied to the main outcome variable.

Estimated quality-adjusted life years (QALYs) gained at the population level, with corresponding 95% CI, as determined using parametric methods and bootstrap techniques. Given the 1-year time horizon, no discount rates will be applied.

Calculation of cost-utility ratio: this is an exploratory objective for which a specific design has not been applied. The cost-utility ratio will be estimated by dividing the total cost by the sum of the potential gains expressed in QALYs. A multivariate sensitivity analysis will be performed in which costs will oscillate within the range of uncertainty of a normal distribution. The benefits (QALYs) will also oscillate within the same range.

## Discussion

This pragmatic clinical trial will involve the participation of FPs from over 50 PC health centres in different geographic areas of Spain, thus ensuring a high level of external validity, given that the PC model implemented throughout the country is relatively homogeneous.

To address the potential contamination among patients of the same cluster, the FP is considered the unit of randomization and the patient the unit of analysis. Still, there is evidence of contamination when healthcare professionals working in the same teams are randomized. To palliate this problem, the following measures will be introduced. First, during the welcome and training session with all participating FPs, we will avoid sharing too many details regarding the complex intervention [45]. Second, participating FPs will be asked to sign a confidentiality agreement once they are randomly assigned to the intervention or control group. Third, the intervention group will be periodically reminded about the importance of avoiding the exchange of any information with other participating FPs during the intervention. Last, by signing the researcher’s commitment, those FP eventually belonging to the control group agree to receive the same training activity once the intervention is over.

Due to the nature of the intervention, it cannot be masked. However, outcome evaluation will be conducted by skilled FPs and pharmacists that are blinded for treatment allocation. The statistician conducting the analysis will neither know to which study arm a given patient has been assigned.

The variability in clinical practice of the different physicians involved and their baseline knowledge on the content covered in the intervention could result in lower than expected differences between groups after completion of the intervention. To address this potential bias, variables related to FPs’ prior training in polypharmacy, multimorbidity, and/or shared decision making will be collected and adjusted for in the multilevel model. Furthermore, the MOOC format of the training material will ensure homogenization of the training received by the FPs. Video technology and online courses have shown to be powerful tools to empower both patients and health professionals and have the potential to significantly improve the delivery of care in an increasingly complex healthcare system [46].

FPs who agree to participate in an experimental study are potentially more interested in the subject, just as patients who agree to participate may share certain common features in terms of health-related awareness and motivation, which can lead to bias. Although better results than those obtained including less selective participants may occur, this phenomenon would have a conservative effect (i.e. it would decrease the magnitude of the difference between the two groups). Moreover, participating FPs could modify or improve their prescribing habits just as a response to their awareness of being observed (Hawthorne effect). This may dilute differences between intervention and control groups regarding the appropriateness of prescription.

Although the analysis of the effect of the intervention on quality of life is non-confirmatory and the expected changes are limited over a short period of time, we consider it essential to include this outcome in the study. Firstly, between-group differences may be detected despite not being highly significant, and secondly, measurement of quality of life changes allows the incorporation of outcome variables reported by patients themselves, and facilitates the calculation of utilities for the cost-utility analysis. This will enable responding to some of the shortcomings and limitations of previous interventions as detected by Patterson et al. [25].

It is essential to provide evidence concerning interventions on PC patients with polypharmacy and multimorbidity, conducted in the context of routine clinical practice, and involving young-old patients with significant potential for preventing negative health outcomes.

## Additional files

Additional file 1: Model of the informed consent completed by participants. (DOC 51 kb)

## Abbreviations

AC: Autonomous community
CPG: Clinical practice guideline
DE: Design effect
FP: Family physician
ICPC: International classification of diseases in PC
MAI: Medication appropriateness index
MOOC: Massive online open courses
PC: Primary care
QALYs: Quality-adjusted life years
