# Supplementary material for: Effectiveness of an intervention for improving drug prescription in primary care patients with multimorbidity and polypharmacy: study protocol of a cluster randomized clinical trial (Multi-PAP project)
Source: Implement Sci. 2017 Apr 27;12:54. doi: 10.1186/s13012-017-0584-x (PMC5406997; doi:10.1186/s13012-017-0584-x)
Supplement: Supplementary file 1 — Model of the informed consent completed by participants. (DOC 51 kb) [file 13012_2017_584_MOESM1_ESM.doc]

**Additional file 1**

**DOCUMENTO DE INFORMACIÓN PARA EL PARTICIPANTE**

**Título de la investigación: Proyecto MULTI-PAP**

**Efectividad de una intervención centrada en el paciente para mejorar la adecuación de la prescripción en pacientes ancianos jóvenes con multimorbilidad y polimedicación en Atención Primaria. Ensayo Clínico por Conglomerados**

**(Expedientes FIS-ISCIII: PI15/00996, PI15/00276 y PI15/00572)**

**1. Introducción:**

Nos dirigimos a usted para invitarle a participar en un proyecto de investigación que estamos realizando en Centros de Atención Primaria de Andalucía, Aragón y Madrid. Su participación es importante para obtener el conocimiento que necesitamos, pero antes de tomar una decisión debe:

- Leer este documento entero

- Entender la información que contiene el documento

- Hacer todas las preguntas que considere necesarias

- Consultar con su médico

- Tomar una decisión meditada

- Firmar el consentimiento informado, si finalmente desea participar.

Si decide participar se le entregará una copia de este documento y del consentimiento firmado. Por favor, consérvelos por si lo necesitara en un futuro.

**2. ¿Por qué se le pide participar?**

Se solicita su colaboración por ser usted un paciente con al menos tres problemas de salud crónicos (multimorbilidad) y que precisa más de 4 medicamentos distintos (polimedicación), siendo atendido por su médico de familia.

En total, en el estudio participarán 500 pacientes con estas características y que, además, tengan entre 65 y 74 años de edad.

**3. ¿Cuál es el objeto de este estudio?**

Cada vez es mayor el número de personas mayores de 65 años, que presentan varios problemas crónicos de salud, para los que necesitan tomar medicamentos. Cada Guía de tratamiento de enfermedad (diabetes, hipertensión, etc) recomienda diferentes fármacos para cada problema concreto. Sin embargo, estas Guías no suelen contemplar que en muchos de estos pacientes coinciden varios problemas crónicos, para los cuales se recomiendan diferentes medicamentos. Existen aspectos que no conocemos bien acerca del efecto global de este conjunto de medicamentos, que en algunos casos podría ocasionar algunos efectos perjudiciales. También sabemos que tener más edad, más enfermedades y más medicación, va empeorando la calidad de vida y la capacidad funcional para mantener actividades comunes de la vida diaria.

Por ello ***queremos estudiar una diferente forma de abordar este problema mediante una entrevista específica entre el médico de familia y su paciente, siguiendo un modelo de atención centrado en el paciente y que valore, no solo los problemas de salud que tiene, sino también sus preferencias para mejorar el resultado final de la medicación.***

Los resultados se medirán a través de un cuestionario de preguntas que usted contestará al inicio y al final del estudio (Cuestionario llamado “MAI”). Al finalizar el estudio se compararán los resultados en el grupo de pacientes que han seguido este modelo de entrevista (grupo de intervención), frente a los resultados en los pacientes que han sido tratados por su médico de la forma habitual (grupo control).

Los pacientes que acepten participar serán incluidos en uno de los dos grupos: de intervención o de control, pero ni el médico ni el paciente eligen el grupo que les corresponde, ya que se decide al azar por los organizadores del estudio. Esta modalidad de distribución al azar, es la mejor manera de que los resultados obtenidos tengan alguna utilidad, es decir, que realmente sirvan para los objetivos del estudio.

Los médicos recibirán formación y entrenamiento específico para poder desarrollar este modelo de entrevista centrada en el paciente.

Estudiamos si este tipo de intervención puede conseguir:

- Mejorar la utilización de medicamentos: utilizar los más adecuados, los que tengan menos efectos secundarios. Y, por otro lado, mejorar el cumplimiento del tratamiento por parte del paciente.
- Mejorar la calidad de vida del paciente.
- Mejorar la utilización de servicios sanitarios.

**4. ¿Qué tengo que hacer si decido participar?**

Dentro del estudio revisaremos su historia clínica. A todos los participantes se les realizarán tres visitas, la primera al inicio del proyecto, la segunda a los 6 meses y la última al año. Las visitas serán realizadas por su médico de familia. Al inicio del estudio, el médico le hará una serie de preguntas sobre sus datos personales, sus enfermedades o problemas de salud y su tratamiento, su calidad de vida, la aparición de efectos adversos a la medicación, y sobre el uso de los servicios de salud: hospitalizaciones no programadas, visitas a servicios de urgencias y a su centro de salud.

El cuestionario de valoración específico para este estudio (“MAI”) lo hará un evaluador externo (una persona diferente a su médico), para lo cual acordaremos una cita con usted.

El seguimiento es muy importante en este proyecto de investigación por lo que, si ha decidido participar, le pedimos que se mantenga en contacto con nosotros si no pudiera acudir a alguna de las citas concertadas, y que nos lo comunique lo antes posible para tratar de conseguir otra cita en la fecha que mejor le convenga.

Recuerde que su participación es voluntaria y si decide no participar esto no afectará a su asistencia sanitaria ni a su relación con su médico de familia ni su equipo.

**5. ¿Qué riesgos o molestias supone?**

Dentro del estudio revisaremos su historia clínica y los medicamentos que toma. Este estudio no necesita practicarle pruebas complementarias como análisis o radiografías. No existe ningún riesgo en la participación en este estudio al no utilizarse ningún medicamento diferente a los que usted ya toma.

Cada entrevista supone una dedicación de unos 30 minutos de consulta, y se harán en el mismo Centro de Salud donde usted es atendido.

**6. ¿Obtendré algún beneficio por mi participación?**

Al tratarse de un estudio de investigación orientado a generar conocimiento, es probable que no obtenga ningún beneficio por su participación, si bien usted contribuirá al avance del conocimiento y al beneficio social: al participar en el estudio usted puede ayudar a mejorar la atención a los pacientes que, como usted, tengan más de dos enfermedades crónicas y consuman un elevado número de medicamentos.

Ni usted, ni los médicos participantes, ni los investigadores del proyecto, recibirán ninguna compensación económica por participar y desarrollar este estudio.

**7. ¿Cómo se van a gestionar mis datos personales?**

Toda la información recogida se tratará conforme a lo establecido en la Ley Orgánica 15/99, de protección de datos de carácter personal. En la base de datos del estudio no se incluirán datos personales: ni su nombre, ni su número de historia clínica ni ningún dato que le pueda identificar. Se le identificará por un código que sólo el equipo investigador podrá relacionar con su nombre.

Sólo el equipo investigador tendrá acceso a los datos de su historia clínica y nadie ajeno al centro podrá consultar su historial.

En cualquier momento puede ejercer el derecho a suspender su participación. A partir de ese momento sus datos dejarán de formar parte del estudio, aunque ya se hubieran obtenido con anterioridad a su decisión de abandonar el estudio. Para ello debe ponerse en contacto con su médico de familia.

Las conclusiones del estudio se presentarán en congresos y a través de publicaciones científicas pero se harán siempre con datos agrupados y nunca se divulgará nada que le pueda identificar.

**8. ¿Quién financia el estudio?**

Este proyecto se financia con fondos públicos: a través de una beca de investigación procedente del Fondo de Investigaciones Sanitarias del Instituto de salud Carlos III (ISCIII), que es el principal Organismo Público de Investigación, que financia, gestiona y ejecuta la investigación biomédica en España.

**9. ¿Se me informará de los resultados del estudio?**

Usted tiene derecho a conocer los resultados del presente estudio, tanto los resultados generales como los derivados de sus datos específicos. También tiene derecho a no conocer dichos resultados si así lo desea. Por este motivo en el documento de consentimiento informado le preguntaremos qué opción prefiere.

**10. ¿Puedo cambiar de opinión?**

Tal como se ha señalado, su participación es totalmente voluntaria, puede decidir no participar o retirarse del estudio en cualquier momento sin tener que dar explicaciones y sin que esto repercuta en su atención sanitaria. Basta con que manifieste su intención a su médico de familia.

**11. ¿Qué pasa si me surge alguna duda durante mi participación?**

En caso de duda o para cualquier consulta relacionada con su participación puede ponerse en contacto con el investigador responsable, D.(Incluir nombre completo del investigador), en el teléfono _______________(indicar teléfono y horario) o por correo electrónico en la dirección _______.

Muchas gracias por su atención, si finalmente desea participar le rogamos que firme el documento de consentimiento que se adjunta.

DOCUMENTO DE CONSENTIMIENTO INFORMADO

Título del PROYECTO:  **Proyecto MULTI-PAP**

**Efectividad de una intervención centrada en el paciente para mejorar la adecuación de la prescripción en pacientes ancianos jóvenes con multimorbilidad y polimedicación en Atención Primaria. Ensayo Clínico por Conglomerados**

**(Expedientes FIS-ISCIII: PI15/00996, PI15/00276 y PI15/00572)**

Yo, .............................................................................. (nombre y apellidos del participante)

He leído el documento de información que se me ha entregado.

He podido hacer preguntas sobre el estudio y he recibido suficiente información sobre el mismo.

He hablado con: ...........................................................................(nombre del investigador)

Comprendo que mi participación es voluntaria.

Comprendo que puedo retirarme del estudio:

1) cuando quiera

2) sin tener que dar explicaciones

3) sin que esto repercuta en mis cuidados médicos

Presto libremente mi conformidad para participar en el estudio**.**

Deseo ser informado sobre los resultados del estudio: sí no (marque lo que proceda)

Doy mi conformidad para que mis datos clínicos sean revisados por personal ajeno al centro para los fines del estudio, y soy consciente de que este consentimiento es revocable.

He recibido una copia firmada de este Consentimiento Informado.

| Firma del participante: |  |
| --- | --- |
| Fecha: |  |
|  |  |

He explicado la naturaleza y el propósito del estudio al paciente mencionado

| Firma del Investigador: |  |
| --- | --- |
| Fecha: |  |
